# Two‐year weight trajectories following completion of a behavioral weight loss maintenance intervention

**DOI:** 10.1002/osp4.491

**Published:** 2021-03-06

**Authors:** Kara L. Gavin, Corrine I. Voils, William S. Yancy, Maren K. Olsen

**Affiliations:** ^1^ Department of Surgery University of Wisconsin School of Medicine and Public Health Madison Wisconsin USA; ^2^ Research Service William S. Middleton Memorial Veterans Hospital Madison Wisconsin USA; ^3^ Department of Medicine Duke University School of Medicine Durham North Carolina USA; ^4^ Center for Health Services Research in Primary Care Durham VA Health Care System Durham North Carolina USA; ^5^ Department of Biostatistics and Bioinformatics Duke University School of Medicine Durham North Carolina USA

**Keywords:** electronic health record, intervention, weight loss maintenance

## Abstract

**Introduction:**

Long‐term effects of behavioral weight loss maintenance interventions need to be assessed in order to understand their durability of effects. This can be evaluated with the use of weights recorded in the electronic medical record. The goal of this study was to use electronic health record (EHR)‐recorded weight to examine outcomes 2 years beyond the completion of a trial in which participants were randomized to receive a weight maintenance intervention or usual care after required initial weight loss.

**Methods:**

Weights collected in the Veteran's Affairs national EHR were obtained for 2 years following trial completion. Outliers and implausible weights were identified and removed prior to analysis. Mixed‐effects models with quadratic time were fit to estimate between‐arm differences in weight change.

**Results:**

Model‐estimated weight at trial completion was 109.7 kg for usual care and 106.8 kg for intervention, estimated difference of −2.9 kg (95% confidence interval [CI]: −8.8, 3.0; *p* = 0.34). Two years later, estimated mean weight collected from (*n* = 211) participants with available EMR weights was 111.5 kg for usual care and 108.0 kg for intervention, estimated difference −3.4 kg (95% CI: −9.3, 2.4 kg; *p* = 0.35).

**Conclusions:**

While not statistically significant, weights from the EHR suggest the possibility of a clinically meaningful difference that should be confirmed by future adequately powered studies.

## 1 INTRODUCTION

To invest in implementation of effective behavioral interventions for long‐term weight loss, understanding the long‐term outcomes of such interventions is necessary. Behavioral weight loss interventions help individuals achieve clinically significant weight loss compared to minimal intervention controls up to 12 months following randomization.^1^ The POWER trial found clinically significant weight loss out to 24 months with continued weight loss support offered to participants in the intervention groups every 3 months.^2^ Additionally, while long‐term weight outcomes out to 15 and 11 years have been assessed in the Diabetes Prevention Program and the Look AHEAD trials, respectively, these interventions required participants be contacted for continued intervention once yearly at minimum postintervention.^3^, ^4^ Thus, questions have been raised about the durability of weight loss after these interventions end. More recent behavioral weight loss trials have included up to 12 months of continued assessment without intervention, finding that many participants begin to regain weight after contact ceases.^1^, ^5^, ^6^, ^7^, ^8^ This work has prompted more focus on strategies to improve weight loss maintenance.

Trials focusing on weight loss maintenance interventions have taken two forms: (1) randomization of participants who recently lost a required amount of weight (either in a trial‐provided program or elsewhere) to one or more weight loss maintenance interventions,^9^, ^10^, ^11^, ^12^ or (2) add maintenance‐focused interventions to the end of a weight loss program without requiring initial weight loss.^13^, ^14^ Most of these maintenance interventions have been 12–24 months in duration, with one trial 30 months in duration.^10^ Due to the prevalence of obesity and the limited resources available to deliver weight management interventions on a population level, it may not be feasible, sustainable, or necessary^15^ to have continued contact over time. Instead it is conceivable—and, indeed, hoped—that participants who learn weight loss maintenance skills will continue to utilize those skills beyond the intervention period. Because the duration of follow‐up periods are primarily determined by grant funding mechanisms, there rarely has been an opportunity to evaluate long‐term outcomes. Nevertheless, the United States Preventive Task Force has recently highlighted the need for examining longer‐term outcomes of these interventions on weight.^16^

In other domains, long‐term outcomes have been examined beyond the trial period using outcomes obtained from the electronic health record (EHR). These include investigations of long‐term clinical and economic effects of diabetes and blood pressure interventions.^17^, ^18^, ^19^ To our knowledge, this method has yet to be used to examine outcome durability after completion of weight loss maintenance trials. The veterans affairs (VA) EHR offers a unique opportunity to examine long‐term weight while reducing loss to follow‐up due to relocation or insurance changes. Both of these situations have been cited as common drawbacks to using EHR data from a typical hospital system.^20^ Through the VA, veterans have insurance coverage expanding many years that is not dependent on employment and are less likely to experience interruptions in insurance coverage due to losing or switching jobs. Even if veterans relocate, as long as they access care through the VA, the EHR will store their health information, thus allowing for follow‐up of long‐term outcomes. The goal of this analysis was to use VA EHR‐recorded weight to examine long‐term weight outcomes 2 years beyond completion of a trial in which veterans were randomized to receive a weight maintenance intervention or usual care after required initial weight loss. Such an investigation will contribute to the sparse literature on durability of weight loss maintenance interventions and offer insights about whether continued contact is required to maintain intervention effects.

## 2 METHODS

The study design and main outcomes have been published.^12^ Briefly, a two‐arm randomized controlled trial tested a primarily telephone‐delivered weight loss maintenance intervention (*n* = 110) against usual care (*n* = 112) in veterans with obesity who had lost ≥4 kg in a study‐provided, 16‐weeks weight‐loss program. The maintenance intervention was designed so that participants would learn and practice maintenance‐specific behavioral skills with an interventionist. These included reflection of satisfaction with outcomes, self‐monitoring, relapse prevention, and soliciting social support. Three in‐person group sessions were held at Weeks 2, 6, and 10 of the intervention with eight individual telephone calls with an interventionist occurring at Weeks 4, 8, 12, 16, 20, 24, 32, and 40.^12^ Phone calls with the interventionist focused on helping individuals with implementation of the four maintenance constructs. Overtime, participants were encouraged to practice these skills on their own and elicit social support from someone in their social network as the frequency of interventionist contact decreased. This allowed the interventionist to check in and troubleshoot challenges to implementing the maintenance‐specific behavioral skills as participants transitioned from depending on the interventionist for supportive accountability.^21^

Randomization was stratified by initial weight loss of <10 kg versus ≥10 kg. In the trial, participants were weighed by study personnel at study entry, following the initial 16‐weeks weight‐loss program (randomization), and 14, 26, 42, and 56 weeks following randomization. The primary outcome of study‐measured weight change 56 weeks following randomization showed a small benefit of the maintenance intervention using a constrained longitudinal analysis model (−1.6 kg; 95% confidence interval [CI]: −0.1, −3.1; *p = *0.04).^12^

For current analyses of long‐term weight loss beyond the end of the trial, we obtained weights from the VA EHR collected at any outpatient visit from the primary completion date to 2 years afterward. Clinical protocols for collecting weight (i.e., with or without shoes and outer layers of clothing and the time of day a patient is weighed, etc.) were not assessed and likely varied.

For this analysis, implausible weights (extreme weights >700 or <50 lbs) were removed. If two or more weights in a single day were recorded, the mean was taken as long as the weights standard deviation was less than two. Thus, only one weight per day was included in the data set. If it the standard deviation was greater than two, clusters of adjacent weights were used to identify the weight that led to the lower standard deviation. Outliers were identified and removed by examining a rolling standard deviation of consecutive weights. Finally, a visual inspection of the weights before and after cleaning was conducted to confirm stable weight patterns.

Mixed‐effects models were fit to estimate between‐arm differences in weight change for 2 years beyond trial completion. Model specification was guided by descriptive analyses and best model fit via the Akaike information criterion index.^22^ Fixed‐effects model coefficients included the main effect for intervention arm, linear and quadratic time terms, and the interactions between intervention and the time terms. Random effects included a random intercept and time. For ease of interpretation, time was coded as the continuous number of months from the trial's primary end point (i.e., time 0, or baseline, for this analysis).

## 3 RESULTS

Among the 222 patients originally randomized in the trial, 211 (*n* = 106 intervention; *n* = 105 usual care) are included in this analysis: six patients in the control arm and five patients in the intervention arm had no EHR weights during the 2 years beyond trial completion. At trial entry, 84% (*n* = 177) of participants were male and 58% (*n* = 120) were White, with a mean age of 61.8 years (SD = 8.3 years). On average, participants had a mean of 6.1 (median of 4, range 1–31) weight measurements included in analyses. Further examination of the data showed that these were spread out fairly evenly throughout the 2‐year period, such that participants had an average of 2.5–3.7 weights at each 6‐months interval.

**FIGURE 1:**
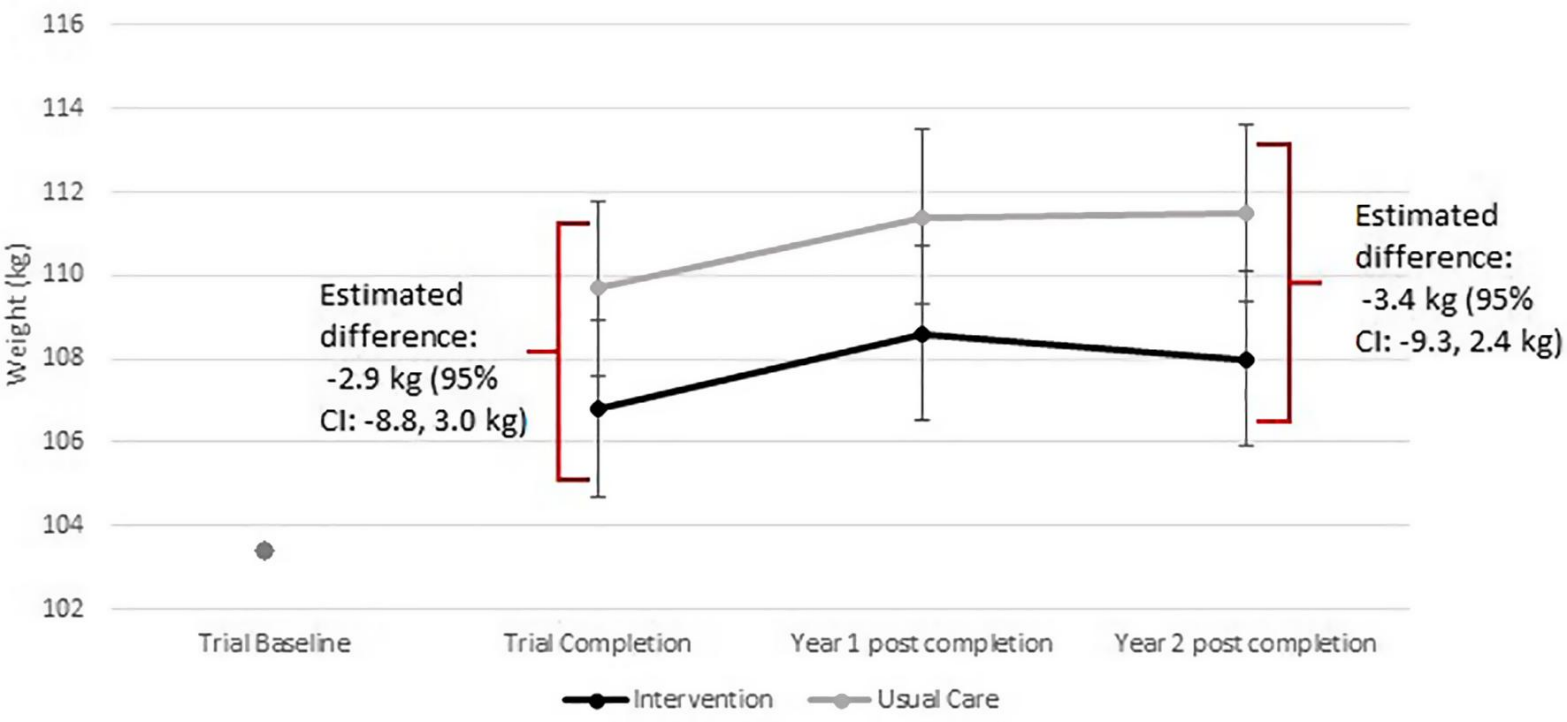
Weight change following the conclusion of a weight loss maintenance intervention. *Note*: Trial baseline weight estimate taken from original study and not included in the statistical models presented here

The estimated weight at trial completion (i.e., baseline for this analysis) was 109.7 kg for usual care and 106.8 kg for intervention, an estimated difference of −2.9 kg (95% CI: −8.8, 3.0). One year beyond trial completion, estimated mean weight was 111.4 kg for usual care and 108.6 kg for intervention participants, estimated mean difference −2.8 kg (95% CI: −8.6, 3.0; *p* = 0.34). Two years beyond trial completion, estimated mean weight was 111.5 kg for usual care and 108.0 kg for intervention participants, estimated mean difference −3.4 kg (95% CI: −9.3, 2.4 kg; *p* = 0.35; Figure 1).

## 4 DISCUSSION

Findings from this current work did not reach statistical significance and highlight the need for further investigations into the long‐term effects of behavioral weight management trials beyond the trial window in order to inform clinical and policy recommendations. This work serves as one example of how future projects may track weight data long‐term. To our knowledge, this study is the first of its kind to report long‐term EHR‐based weight data beyond completion of a weight loss maintenance trial. The original trial was powered to detect a clinically significant difference between arms of 3.5 kg at 12 months, as this was considered clinically significant. One and two years later, differences of approximately 3 kg suggest a clinically meaningful,^23^ enduring benefit of the intervention. Though not significant, the between‐group difference was clinically meaningful 24 months following trial completion and is similar in magnitude to effect sizes found at trial completion in other successful weight maintenance focused interventions.^11^, ^12^, ^14^, ^24^ The between‐arm differences were not statistically significant owing to the wider standard errors associated with EHR weights. This is due to both a larger standard deviation and lack of a fixed sample size at the estimated time points, unlike the fixed time points and in person weights typically measured in intervention trials. These factors must be taken into account when designing trials that use EHR data to test durability of intervention effects.

This study underscores the EHR as an effective tool for tracking weight changes following trial completion. Although participants do not have weight measures taken at uniform time periods, statistical models can utilize all weights, collected at varying points in time, to estimate mean weight values at specific time points. While similar investigations outside the VA healthcare system are possible given the widespread adoption of EHR systems, the use of the VA EHR is unique in its ability to avoid selection biases from loss to follow‐up that may occur from individuals seeking care from multiple health systems. Utilization of EHR data is increasingly common in pragmatic trials. A major advantage is reducing patient burden from attending study visits. Using EHR data, however, poses unique challenges. For example, there will be suspected data entry errors and/or outlier values, necessitating establishment of parameters to identify such weights. Additionally, weight was taken during clinical encounters on different scales, not using a study protocol requiring removal of outerwear and shoes adding to variability of the recorded weight. In this analysis, implausible weights were identified based on a rule that weights greater than 700 lbs or less than 50 lbs should be excluded. As mentioned, outliers were identified using a rolling standard deviation and visual inspection errant values. Finally, power calculations for such studies need to account for the larger anticipated standard deviation due to variable weight measurement protocols in clinics and unequal timing of measurements.

In conclusion, although our results were not statistically significant, they suggest the possibility of clinically meaningful, long‐term effects of a weight loss maintenance intervention. Future research is needed to confirm these findings in sufficiently powered studies.

## CONFLICT OF INTERESTS

The authors declare no conflict of interest.

## AUTHOR CONTRIBUTIONS

*Conception and design*: Kara L. Gavin, Maren K. Olsen, William S. Yancy, Corrine I. Voils. *Data analysis and statistical expertise*: Maren K. Olsen. *Drafting of the article, critical revisions, and final approval*: Kara L. Gavin, Maren K. Olsen, William S. Yancy, Corrine I. Voils.
